# The Analytical Framework of Clinical Trials Evaluating Clinical Outcomes of Artificial Intelligence-Based Digital Health Interventions: A Systematic Literature Review

**DOI:** 10.3390/jmahp14030038

**Published:** 2026-07-01

**Authors:** Vladimir Zah, Dimitrije Grbic, Carl Asche, Filip Stanicic

**Affiliations:** 1 Health Economics and Outcomes Research Department, ZRx Outcomes Research Inc., 3450 Cawthra Road, Mississauga, ON L5A 2X7, Canada; dgrbic@outcomesresearch.ca (D.G.); fstanicic@outcomesresearch.ca (F.S.); 2 Department of Pharmacotherapy, College of Pharmacy, University of Utah, 201 Presidents’ Cir, Salt Lake City, UT 84112, USA

**Keywords:** digital health tools, artificial intelligence, clinical efficacy, clinical trials

## Abstract

**Introduction:** This systematic literature review (SLR) provides an analytical framework for clinical trials evaluating clinical outcomes of artificial intelligence-based digital health interventions (AI-DHI). **Methods:** The SLR was conducted in accordance with the PRISMA guidelines. Search was conducted (September 2025) in PubMed and Embase. Population included patients using AI-DHI. Only clinical trials exploring clinical outcomes, written in English, were considered. NICE checklist was used to assess studies’ quality. Results were analyzed descriptively. **Results:** Final sample had 84 studies, with metabolic (28.6%), musculoskeletal (20.2%), and mental health disorders (19.0%) as the most common indications. Most studies (75.0%) were controlled, parallel-group trials with 2+ arms, mostly comparing AI-DHI with standard-of-care or waitlist. Although type of intervention often precludes blinding (64.3% were open-label), a double-blinding is strongly recommended (only 6.0%). Only 9.5% of studies were conducted at multiple sites across different countries. Dropout rates in the total sample and each study arm should be <20% at all endpoints (64.3%). Statistical tests were used based on the outcome measures. The small sample sizes and limited generalizability of findings were reported as the main limitations. **Conclusions:** This SLR emphasized current methodological gaps and an urgent need for unified global guidelines. Standard SLR limitations apply to this research.

## 1. Introduction

Digital health intervention (DHI) is a technology that leverages computing platforms, connectivity, software, and/or sensors for use in healthcare or related services. DHI is a broad term encompassing categories such as mobile health (mHealth), telehealth and telemedicine, and health information technology. The tools include technologies intended for use as a medical product, as part of a medical product, or for developing and studying other medical products. By connecting people, information, and technology, these advancements are improving both the delivery of care and overall health outcomes [1,2].

The integration of DHIs is transforming healthcare into a more comprehensive and patient-centric model. The use of these tools provides a holistic perspective on patients’ health for both clinicians and patients, enabling early diagnosis and personalized management. It is also easier to track health status and to prevent disease-related events promptly. From the healthcare system’s perspective, DHIs offer practical solutions to improve access to healthcare, reduce overall costs, and enhance the quality of provided services [1]. The use of technology for health-related purposes in the United States (US) has increased drastically over the years, regardless of the clinical and sociodemographic population determinants [3]. Still, there are multiple barriers to the successful implementation and use of DHIs in healthcare, including infrastructure and technical barriers, personal and psychological issues, and concerns about increased working hours or workload among healthcare professionals. Appropriate training, educational programs, multisector incentives, and perception of DHI effectiveness are necessary measures to overcome these barriers [2].

Regulatory requirements for demonstrating the value of DHIs differ from those for conventional health technologies in certain ways. Health technology assessment (HTA) for traditional interventions is a multidisciplinary process that evaluates all available evidence on the product’s efficacy, safety, cost-effectiveness, budget impact, and the ethical and legal aspects of its use, in comparison with national and international standards. In addition to these standard requirements, HTA of DHIs typically involves assessing specific domains, such as usability, acceptability, data security, interoperability, and technical considerations [4,5,6]. HTA requirements vary between healthcare systems and also depend on the type of DHI [4,7]. For example, certain guidelines provide a hierarchical classification of the required level of evidence appropriate for the specific DHI type. DHIs used directly for diagnostic or treatment purposes should provide high-quality clinical trial evidence when compared with a suitable comparator from real-world clinical practice. On the other hand, the requirements for simpler technologies developed solely to communicate information or provide support services in healthcare delivery are lower in the hierarchy. The focus of HTA is primarily on the credibility, quality, and accuracy of the content provided [7].

The rapid advancement of artificial intelligence (AI) in recent years has significantly impacted the healthcare sector. Implementing an AI component into DHIs extended the application and benefits of these tools. AI is enhancing DHIs for diagnostic and imaging procedures, providing real-time intraoperative support, aiding many drug discovery segments, guiding treatments with improved personalization, and facilitating the integration of telehealth and other patient-monitoring tools. However, the successful integration of AI-based DHIs comes with multiple new challenges. These challenges primarily concern ensuring patient data security, preventing algorithmic bias, ensuring algorithmic transparency, addressing complex ethical issues, and more [8,9].

The successful implementation of innovative AI-based DHI is contingent on providing high-quality evidence of the intervention that meets the specific national and/or international requirements for the respective device type. A thorough plan for performing a well-designed clinical trial is the initial and most crucial step of the HTA process. The study design should be developed in accordance with both regulatory agency guidelines and standard research practices for similar technologies.

The main objective of this systematic literature review (SLR) is to provide an analytical framework and global methodological recommendations based on clinical trials evaluating the clinical outcomes of AI-based DHIs. The SLR results should guide researchers in designing clinical trials to synthesize evidence on the efficacy of such devices, in line with current research practice.

## 2. Materials and Methods

### 2.1. Data Sources and Selection Criteria

The key literature databases for the SLR were PubMed and Embase. The search was conducted in September 2025. The protocol for this SLR was not previously published. As the primary outcomes were related to methodological approaches and clinical trial design, no time limitations were applied when collecting all relevant publications evaluating the clinical efficacy of DHIs in existing literature. In addition, a hand search was conducted across publicly available domains (e.g., Google Scholar) and the reference lists of other published reviews to ensure that all relevant studies were included. Only clinical trials were considered, while other research designs were excluded. Detailed selection criteria are shown in Table 1.

### 2.2. Search Strategy

Search queries (Supplementary Table S1) were constructed to capture clinical trials evaluating clinical outcomes of AI-based DHIs. The criteria used to define clinical outcomes were not included in the queries because database search algorithms lacked sufficient sensitivity. Therefore, these criteria were considered during the title, abstract, and full-text screening phases. The search was based on the research question defined by Population, Intervention, Comparators, Outcomes, and Study design (PICOS) criteria (Table 2).

### 2.3. Literature Review and Extraction

The review was conducted in accordance with the Preferred Reporting Items for Systematic Reviews and Meta-Analysis (PRISMA) guidelines [10]. Two independent reviewers performed the database search, abstract and title, and full-text screening. All disagreements were resolved between them after each screening phase. Predefined extraction tables were used for data collection and evidence summary.

### 2.4. Quality Assessment

The quality appraisal checklist for quantitative studies proposed by the National Institute for Health and Care Excellence (NICE) was used by two independent reviewers to assess and grade each study included in the extraction process (Supplementary Materials Table S2). High accuracy and agreement between reviewers were ensured by a consistent study evaluation during the double assessment of a random 10% sample (10 studies).

### 2.5. Data Synthesis

Upon the completion of all previous phases, the results were reported and discussed in the executive summary format. The qualitative summary was conceptualized based on the disease area and geographic regions of published clinical trials, further stratified into groups.

## 3. Results

**Figure 1:**
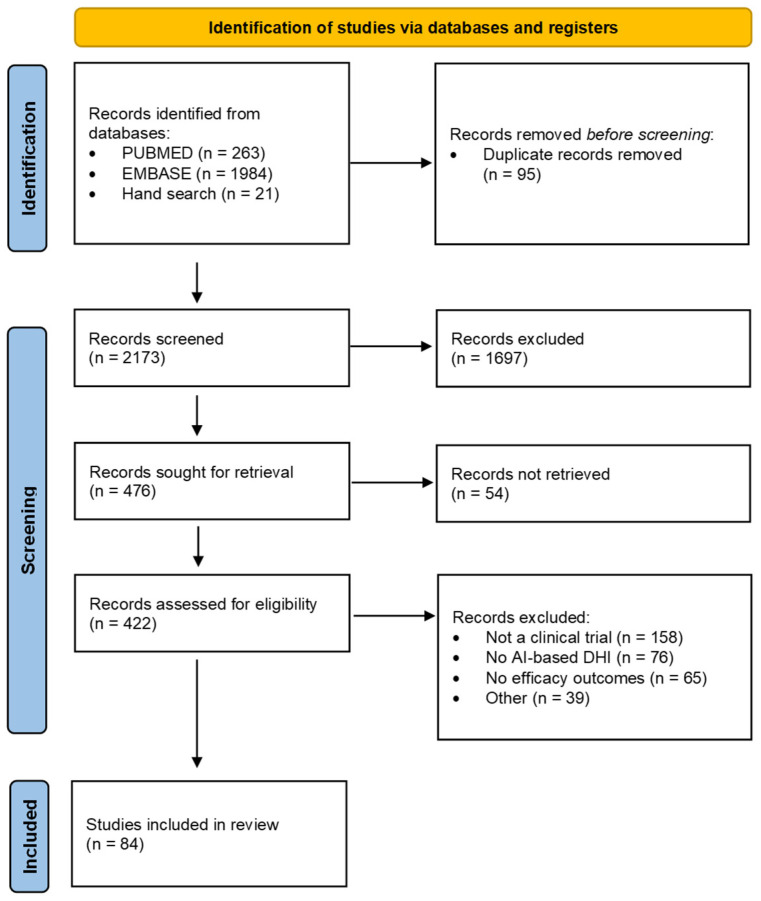
PRISMA flow chart.

A literature search with predefined queries yielded a total of 2247 hits. After removing duplicate studies (*n* = 93), 2152 studies were reviewed during the title and abstract screening process. There were 1751 records excluded during this phase, leaving 401 studies for full-text screening. After the full-text review, 75 studies were eligible for quality assessment and data extraction. Of those studies that were excluded, 157 studies were not clinical trials, 76 studies had irrelevant outcomes, 73 studies assessed DHIs but without an AI component, 18 studies were clinical trial protocols or secondary analyses of clinical trials, and 2 studies did not assess DHIs. In addition, 21 studies were identified via hand search and deemed eligible according to the predefined criteria. During the quality assessment process, 12 studies were excluded (mainly due to insufficient data or poor study quality), resulting in a final sample of 84 studies qualified for the data extraction and evidence synthesis. PRISMA flow diagram of the literature review is presented in Figure 1.

**Figure 2:**
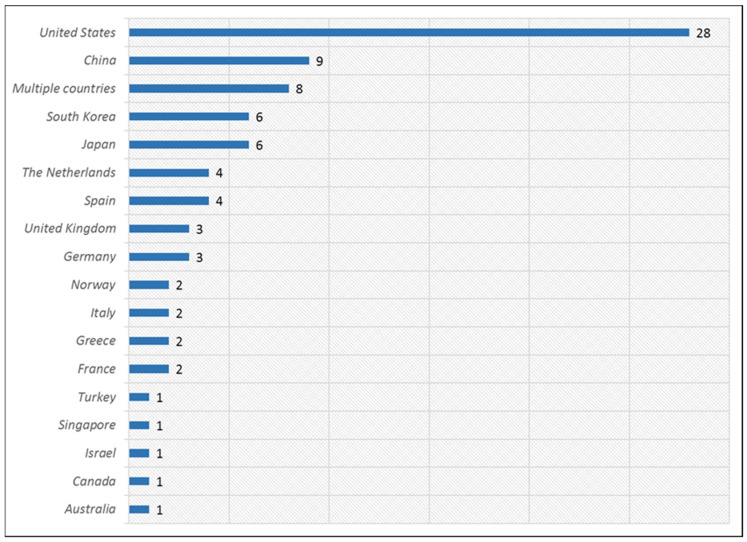
Number of studies stratified by country.

The highest number of studies was conducted in the US (33.3%) and China (10.7%), with 9.5% of multicenter studies conducted in multiple countries (Figure 2).

**Figure 3:**
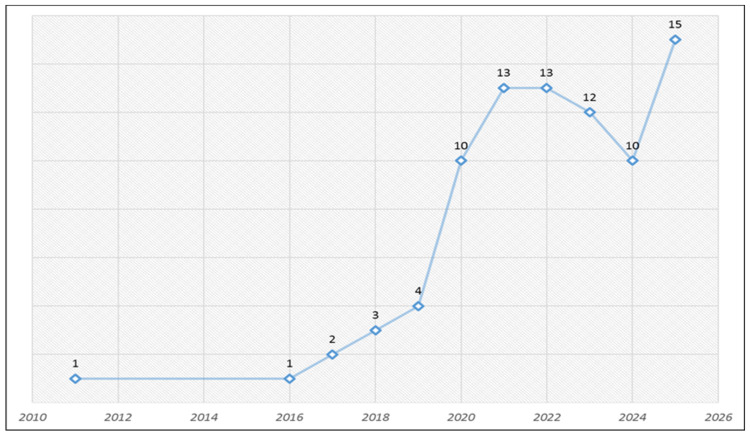
Number of studies stratified by publication year.

The increasing publication trend was noted from 2011 to 2025, with most studies (75.0%) conducted after 2020 (Figure 3).

**Figure 4:**
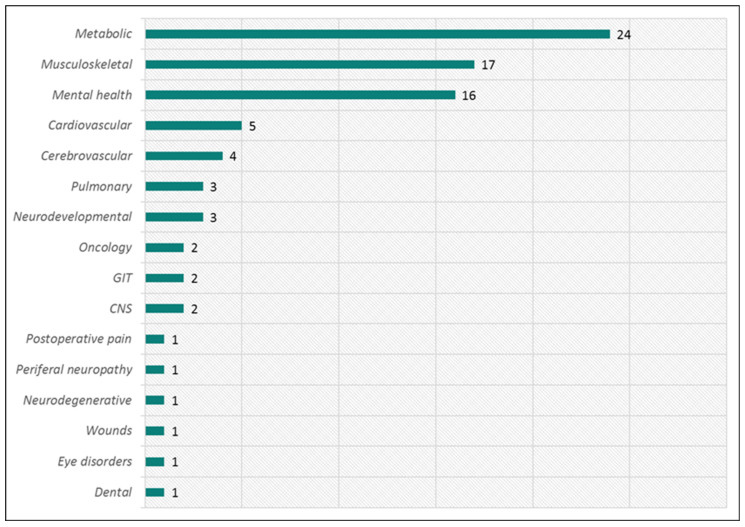
Number of studies stratified by disease area. Abbreviations: GIT—Gastrointestinal tract; CNS—Central nervous system.

It was observed that 24 studies evaluated AI-based DHIs in metabolic disorders, 17 in musculoskeletal disorders, and 16 in mental health (Figure 4).

The majority of clinical trials (82.1%) were controlled studies (75.0% parallel and 7.1% cross-over), while 17.9% were single-arm clinical trials. Most of the extracted clinical trials were open-label (64.3%), with only 13.1% blinded, and blinding was not reported in 22.6%. Most of the included clinical trials were conducted in a single country (90.5%), of which 28.6% were multicenter studies. The authors performed power analyses in 60.7% of clinical trials to determine a reliable sample size capable of detecting clinically and statistically meaningful differences in measured outcomes. Dropout rates below 20% were observed in 64.3% of clinical trials. The follow-up duration varied across studies and depended on the therapeutic indications and the type of outcomes investigated. It was estimated that 61.9% of clinical trials had follow-up durations ≥12 weeks, with only one study [11] in which the follow-up duration was not clearly defined. The list of all studies included in the evidence synthesis, along with their corresponding design characteristics, is presented in Table 3.

Defined with the PICOS criteria, all included clinical trials evaluated the clinical efficacy of AI-based DHIs. Various relevant clinical outcomes were assessed in these trials, depending on the disease indication and primary objectives. In addition, 34.5% of clinical trials assessed patients’ quality of life, whereas patient satisfaction was investigated in 17.9% of trials. Safety outcomes were evaluated in 21.4% of studies, economic outcomes in only 10.7%, and compliance and adherence measures in 20.2% of studies. DHI’s usability, feasibility, and acceptability were evaluated as secondary outcomes in a small proportion of clinical trials (11.9%, 4.8%, and 6.0%, respectively). The list of all additional outcomes evaluated in the clinical trials of AI-based DHIs is presented in Table 4.

Regression models were employed in the majority of clinical trials (44.0%) to estimate the effects of intervention on study outcomes. It was demonstrated that between-group statistical comparisons (primarily performed independent T-test, chi square test, Fisher exact test, or non-parametric Mann–Whitney U test and Wilcoxon rank test) were performed in 57.1% of clinical trials, while within-group comparisons (i.e., estimating the level of changes from baseline to follow-up endpoint) were performed in 13.1% of clinical trials. The Shapiro–Wilk and Kolmogorov–Smirnov tests were the most commonly used to assess data normality, with 14.3% of included clinical trials employing them. Furthermore, the analysis of variance and the analysis of covariance were performed in 27.4% and 11.9% of clinical trials, respectively. It was observed that 7.1% of clinical trials did not provide any statistical analyses to support their findings. The list of all clinical trials and statistical approaches used in these studies is presented in Table 5.

Limitations of these clinical trials varied and depended on the predefined population characteristics, outcomes of interest, DHI performance, and follow-up duration. The main study constraints included limited generalizability of the study findings due to the small sample sizes in these clinical trials, low digital literacy, and technical issues and requirements related to DHIs implementation. Many clinical trials reported potential bias in the collection of study outcomes because they relied on self-reported measures completed by patients via the investigated DHIs. In addition, the observational periods in most of these clinical trials were not long enough to assess the long-term effects of DHIs. Given the nature of clinical trial design and the strictly controlled observational environment, the effects of DHIs should be re-evaluated in real-world settings after implementation in healthcare systems.

## 4. Discussion

This SLR summarized the key characteristics of clinical trials evaluating the efficacy of AI-based DHIs. Clinical trial design should follow the core recommendations from the central regulatory bodies to provide the highest level of evidence. Despite regulatory bodies giving clear guidelines on the structure of standard clinical trials, AI-based DHI studies varied slightly. This SLR reported that 64.3% of clinical trials were open-label, with complete awareness of the intervention and control arms among both healthcare professionals and study participants. In comparison, 22.6% of trials did not report the level of blinding. Double blinding is the gold standard in clinical trial design, preventing the effects of participants’ expectations about the intervention’s efficacy and of researchers’ subjectivity during observations. The lack of blinding could result in biased study findings, potentially overestimating the intervention’s effects. The small proportion of blinded trials was noted in this SLR due to the nature of the DHIs investigated, as participants were trained to use their digital health devices before the trials began, thereby precluding masking of the randomization process. Conducting clinical trials at multiple medical centers is required to ensure a more heterogeneous population and to improve the generalizability of study findings. This SLR reported that only 28.6% of studies were multicenter, underscoring the need for more trials conducted across multiple healthcare institutions. Power analysis was performed in more than half of the included trials (60.7%) as part of the statistical analysis plan, ensuring an appropriate sample size capable of yielding clinically meaningful outcomes and detecting statistically significant differences between study cohorts with high power. However, in 39.3% of AI-based DHI trials, participant recruitment was not conducted according to the predefined statistical plan, indicating insufficient sample size and a lack of reliability of the findings. According to the NICE quality appraisal checklist for quantitative studies, a dropout rate below 20% was considered acceptable. Most AI-based DHI clinical trials (64.3%) had satisfactory dropout rates according to the NICE checklist. Still, almost one in three studies reported a high proportion of participants who left before the study’s completion. Because the effects of AI-based DHIs on clinical efficacy were the primary outcomes across all included clinical trials, the authors noted that only 34.5% of trials evaluated the impact on participants’ quality of life, and even fewer (17.9%) considered patient-reported outcomes such as patient satisfaction. Patient-reported outcomes should be evaluated as key aspects of DHIs, indicating the advantages and obstacles for end users. The effects of AI-based DHIs may be strongly influenced by patients’ attitudes, knowledge, and acceptance levels. Although only 7.1% of trials did not provide statistical procedures to support their findings, the study results should be strengthened by including between-group or within-group comparisons using appropriate statistical tests in all clinical trials, thereby ensuring the generation of robust evidence. The standard SLR limitations apply to this research. Only two literature databases were used for the clinical trials search, including only full-text, publicly available articles. The results are descriptive; hence, no statistical methods were applied, and quantitative summaries of clinical trial results were not performed.

Several SLRs focusing on clinical trials of AI-based DHIs have been published in recent years, aiming to present the current state of research practice in this field and to enhance understanding of such interventions. The SLR conducted by Lam et al. [95] investigated the performance of AI-assisted digital tools in randomized clinical trials. The review included 39 studies across 13 disease groups. Most frequently, studies were conducted in the US (33%). An increasing trend in the publication of clinical trials using AI-assisted digital tools was observed. The included studies were mostly single-center (59.0%), open-label (41.0%), and parallel-group (79.5%). Double blinding was successfully applied in only 10.3% of trials. The most frequent AI intervention subtype was a convolutional neural network (43.6%). Treatment response, as a clinical endpoint, was reported in only 33.3% of studies. Outcome measures varied across trials, but positive results favoring AI-assisted digital tools were observed in 76.9% of them. The main limitation across clinical trials was the limited generalizability of findings, primarily due to small sample sizes (84.6%) and single-center designs (56.4%). Power analysis results were not reported in 20.5% of trials, and 17.9% of trials were underpowered. Using the Cochrane risk-of-bias tool, a low overall risk of bias was estimated in only 20.5% of included studies [95]. A similar analysis was performed by Han et al. [96], who conducted a scoping review with a systematic literature search that included randomized clinical trials evaluating AI interventions in clinical practice. A total of 86 unique clinical trials were included in this review. The most frequent disease area was gastroenterology (43%). Most studies were conducted in a single country (92%), primarily the US (31%) and China (30%). Also, most studies were single-center trials (63%). The use of Consolidated Standards of Reporting Trials-Artificial Intelligence (CONSORT-AI) was reported in only 19% of trials. The most common type of intervention was a deep learning system for medical imaging (69%). The most frequent comparison was between AI-assisted and unassisted clinicians (67.4%). The primary endpoints pertained primarily to diagnostic performance and accuracy (54% of trials). Other commonly reported primary endpoint categories included care management (21%), patient behavior and symptoms (17%), and clinical decision-making (8%). Statistically significant improvement or non-inferiority of AI-based DHIs was reported in 81.4% of studies. Statistical tests varied based on the outcome measures and data characteristics [96]. The findings of our study are in line with the published literature. Although our SLR includes the most recently published AI-based DHI clinical trials and primarily focuses on clinical outcomes, similar flaws have been observed across the included trials. Most published AI-based DHI clinical trials were open-label, which may explain the intervention type. Double-blind trials may not be feasible for all types of interventions, but they are preferred when possible. An open-label design is a potential source of bias and negatively impacts the reliability of findings. On the other hand, this study differs from other reviews in its systematic approach and inclusion of the most recently published literature on AI-based DHIs. As the number of publications in this field of medical research has increased substantially over the years, many reviews conducted prior to 2025 are already outdated. Also, many researchers use simpler search strategies (a single database, limited search queries, multiple filters, etc.) or literature review methodologies (targeted, scoping, etc.) due to the large number of studies captured. A general summary of efficacy outcomes is also being forced, despite substantial heterogeneity among the included trials. The novelty of the evidence from our study stems from a comprehensive search strategy, a scientifically well-established systematic literature review approach, and a sole focus on clinical trial design and methodology to develop a basis for an analytical framework for improving future clinical trials of AI-based DHIs.

Additionally, many trials are either underpowered or fail to report power analyses. Estimating the required sample size for pilot studies is not necessary, whereas it is mandatory when conducting clinical trials. It should be clearly stated whether the power analysis was performed, regardless of the results. Conducting clinical trials in multi-center and multi-country settings also significantly contributes to the generalizability and reliability of findings. Yet most published clinical trials were conducted in single-center, single-country settings. Hence, the main study limitations were similar among the included studies. The most frequently reported limitation was limited generalizability, often stemming from small sample sizes, short follow-up periods, and single-center designs. Technical issues with devices and DHI implementation were also common, largely attributable to the low digital literacy of the enrolled populations. Furthermore, these interventions typically rely on patient-reported outcomes, which introduces a risk of data collection bias. As these challenges are inherent to AI-based DHI trials and difficult to avoid due to the nature of the intervention, their benefits should be validated using real-world data following implementation in clinical practice.

The rapid progress and development of AI-based DHIs in recent years have raised the need for rigorous regulatory changes worldwide. The current HTA methodologies are unable to fully capture the real added value of DHTs for patients, professionals, and healthcare systems. National and international HTA agencies need to redefine existing frameworks and precisely define the value assessments of such devices to address all relevant issues related to their use in clinical practice. European Digital Health Technology Assessment (EDiHTA) is an ongoing project that brings together all relevant European Union (EU) stakeholders to develop the first flexible, inclusive, validated, and ready-for-use European HTA framework. The framework will apply to all DHI types (including those with an AI component), territorial levels (national, regional, and local), and perspectives (payer, hospital, society, etc.). Piloting will be conducted in real healthcare settings at five major EU hospitals. The EDiHTA framework will be based on a holistic approach that involves all stakeholders to build consensus at the national and EU levels. It will harmonize existing HTA frameworks and optimize DHT assessment processes [97].

The Food and Drug Administration (FDA) also released a framework specifically for AI-based DHIs [98]. The document provides general information and submission recommendations relevant to the US market access for these devices. Manufacturers are required to provide a comprehensive device description that includes a clear explanation of the intended use and users, the device’s inputs and outputs, the clinical workflow, the degree of automation, and the integration of AI with other device components. The user interface must be transparent with concise instructions on how to use the device, model performance data (including across population subgroups if available), and known risks and limitations. The risk assessment and management file should include thorough information on the identification and control of risk factors, such as model opacity, user misunderstanding of outputs, algorithmic bias, and data privacy threats. Transparency involves ensuring that important information is both accessible and functionally comprehensible, while AI bias refers to a potential tendency to produce incorrect results in a systematic or unforeseeable way. Data management (collection, cleaning, annotation, and storage) and dataset descriptions (development and validation) should be explained in detail.

Additionally, cybersecurity protections must address threats unique to AI models. Development documentation must describe the model’s architecture, features, development process (e.g., training methods and hyperparameters), and any pre-processing or post-processing steps, enabling a competent practitioner to understand and replicate the model’s construction. Rigorous performance validation requires objective evidence of clinical efficacy and safety for the intended use. This includes pre-specified study protocols, statistical analysis plans, primary and subgroup analyses, and, where appropriate, human factors validation to assess human-device interaction. FDA clarifies that the pre-specification protocol and statistical analysis plan (including sample size justification and data handling plans) must be finalized before the validation step to avoid bias from post hoc analysis. Validation must strictly adhere to the pre-specified plan and any deviations from the research protocol must be justified. Blinding is essential for AI-based DHIs developed for diagnostic purposes, while for therapeutic devices, it may not always be feasible. However, the FDA still considers a lack of blinding as a potential source of bias in clinical trials. If possible, repeatability (same operator and/or device) and reproducibility (different operators and/or devices) studies should be conducted to assess the robustness of the device’s output with respect to potential measurement variability. Primary endpoints must be clearly defined and clinically justified, with confidence intervals reported alongside point estimates.

All aspects of the AI-based DHI should be considered when defining study endpoints. For example, the FDA recognizes that not every diagnostic device requires precision study, as some devices may be too harmful to patients if used repeatedly. If the AI model includes quality control steps, such as discarding low-quality data, a sensitivity analysis must be conducted. This will ensure that the quality control algorithm does not unfairly exclude challenging cases by misclassifying them as low-quality inputs, thereby overestimating the reported model performance. FDA acknowledges the importance of a Total Product Lifecycle (TPLC) approach for AI-based DHIs. TPLC entails that submission evidence for market access is not sufficient, but it is also necessary to perform post-marketing monitoring to ensure further device development and improve safety and effectiveness throughout the device lifecycle. A post-marketing performance monitoring plan should be developed to proactively monitor the device’s use and performance in real-world clinical practice. This plan should outline methods for detecting and addressing performance degradation resulting from factors such as data drift, changes in the target population, or shifts in clinical practice. In addition to post-marketing surveillance, future studies should place greater emphasis on real-world validation using data from routine clinical practice, including electronic health records, administrative claims databases, and patient registries. Real-world evidence could complement findings from controlled clinical trials by evaluating device performance, effectiveness, and safety in broader and more diverse patient populations, thereby improving the generalizability and applicability of study findings. A final, comprehensive, and publicly available submission summary covering all these aspects must be provided to foster public trust. Yet the FDA explained that AI-based DHIs are not distinguishable from DHIs without AI components, sharing the same intended use in terms of clinical safety and efficacy requirements [98]. The proposed recommendations are designed to be broadly applicable to AI-based DHIs. Still, additional considerations and requirements may apply depending on device characteristics, intended use, and the type of AI component.

Although the FDA framework for AI-based DHIs did not provide detailed guidance on designing clinical trials, it suggested following another FDA guideline for designing pivotal clinical investigations for medical devices in general [99]. Based on the recommendations, clinical trials should be conducted in a reliable population of interest to reduce bias, which may otherwise lead to incorrect determinations of safety and effectiveness. A method of participant selection should provide adequate assurance that the subjects are suitable for the study, diagnostic criteria of the condition to be treated or diagnosed, confirmatory laboratory tests where appropriate, and evidence of susceptibility and exposure to the condition against which prophylaxis is desired (in the case of a device to prevent a disease or condition). In addition, for studies with two or more groups, participants should be assigned in a manner that minimizes potential bias and ensures comparability across cohorts with respect to pertinent variables such as sex, disease severity or duration, and use of therapy other than the test device. Manufacturers should select subject enrollment sites appropriate for the device’s intended use. Single-center studies may be a helpful starting point for evaluating the initial feasibility of a new device, as they are logistically easier to coordinate, less resource-intensive, and typically involve a more homogeneous subject population with fewer confounding variables. Rather, the evaluation of the safety and efficacy of an investigational device typically relies on demonstrating generally consistent results across multiple study sites within a larger multicenter study. Device performance and clinical outcomes should be objectively measured with minimal bias. The endpoints, outcomes, or measurements should provide sufficient evidence to characterize the device’s clinical benefits for the intended use. Furthermore, greater use of patient-reported outcomes should be encouraged in future evaluations of AI-based DHIs. Patient-reported outcomes provide direct insight into patients’ symptoms, functioning, health-related quality of life, and treatment experiences, capturing aspects of intervention benefit that may not be adequately reflected by traditional clinical or performance-based endpoints. When collecting self-reported outcome measures, it is essential to select a scoring tool that is validated for the target population and condition and is consistent with the intended use. Instruments should be fit for purpose and capable of reliably capturing clinically meaningful changes in outcomes that are relevant to patients. The use of robust and interpretable patient-reported outcome measures may improve patient-centeredness and clinical relevance of research, strengthening the value of AI-based DHI evidence for HTA and reimbursement decisions. It is strongly suggested that subjects, investigators, and third-party evaluators be blinded during the trial to avoid potential bias. In some clinical outcome device studies, particularly those that are highly invasive or in which device treatment is compared with medical therapy or surgical intervention, it may be impossible to blind subjects or investigators to the intervention assignment. However, even if blinding is inconvenient or complex, the FDA recommends considering and attempting it when feasible [99].

The high heterogeneity in trial designs, endpoints, and statistical approaches observed in this SLR depicts an urgent need for indication-specific methodological consensus in this field of medical research. Developing a general clinical trial framework for all AI-based DHIs may not be appropriate, given their vastly different intended uses, which directly affect the technology design, data collection, and outcome assessment. For example, in trials of diagnostic AI-based DHIs, the designs must prioritize diagnostic accuracy measures (sensitivity, specificity, and predictive values) in representative, multi-center cohorts. For continuous monitoring or self-management (e.g., diabetes or mental health), longer-term randomized controlled trials are preferable, emphasizing patient-relevant endpoints (e.g., HbA1c, quality of life, or hospitalization rates) alongside adherence and engagement measures. For treatment recommendation algorithms, adaptive platform designs that incorporate dynamic treatment regimens and balance efficacy and safety are most appropriate for capturing overall clinical benefit. Hence, a suitable solution may be to develop indication-specific, AI-based DHI frameworks that define minimal methodological standards for devices based on their intended use. In this scenario, all relevant details of AI-based DHI and clinical trial designs and their alignment with regulatory requirements and modern research practices would be discussed in a mandatory multi-stakeholder consensus involving manufacturers, regulatory, and HTA bodies in earlier development stages. Such a tailored approach would ensure that generated evidence is not only scientifically valid but also legally acceptable, ultimately accelerating patient access to safe and effective AI-based DHIs.

## 5. Conclusions

This SLR provides a comprehensive overview of clinical trial designs evaluating the clinical efficacy of AI-based DHIs across therapeutic areas. The reflection on the official guideline recommendations for DHI clinical trial development and the analytical framework observed in the public domain is also provided, emphasizing the current methodological gaps in this field of research.

Our findings revealed insufficient alignment between the previously conducted trial designs and the upcoming requirements of US and EU regulatory agencies, particularly regarding data sampling, population characteristics, digital literacy, outcome measurement, safety, and the AI performance stability matrix. Future clinical trials evaluating AI-based DHIs should be designed and conducted in alignment with regulatory requirements and emerging best practices in medical research, particularly in study design, outcome assessment, and statistical methods.

There is an urgent need for unified and clear global guideline recommendations that would benefit both regulatory bodies and AI-based DHI manufacturers. This analytical framework could serve as a guide for future research on AI-based DHIs, ensuring reliable, robust evidence and facilitating implementation in clinical practice. In order to further improve the implementation of AI-based DHIs into modern healthcare, the misalignment between trial requirements and research practice must be resolved by developing indication- and device type-specific methodological standards through mandatory early dialogues between manufacturers, regulators, and payers, ensuring that the generated evidence is not only scientifically credible but also sufficient for both market authorization and reimbursement decisions.

## Figures and Tables

**Table 1:** Study selection criteria.

Inclusion Criteria	Exclusion Criteria
1. Studies evaluating AI-based digital health interventions 2. Studies with clinical outcomes of interest (i.e., efficacy, safety, and patient-reported outcomes) 3. Publications written in English	1. Studies evaluating the accuracy of AI-based DHIs 2. Systematic and narrative reviews 3. Direct and indirect treatment comparisons 4. Retrospective, cross-sectional, case-report, and case-series studies 5. Surveys, physician interviews, and questionnaires 6. Preclinical, in vitro, animal, molecular, and genetic studies 7. Guidelines, books, editorials, comments, replies, and letters

Abbreviations: AI—Artificial intelligence; DHIs—Digital health interventions.

**Table 2:** PICOS domains defining the research question for the SLR.

PICOS	Description
Population	Patients diagnosed with any disease
Intervention	AI-based DHIs
Comparators	Any intervention
Outcomes	1. Efficacy
	2. Safety
	3. Patient-reported outcomes
	4. Other outcomes
Study Design	Clinical trials

Abbreviations: AI—Artificial intelligence; DHIs—Digital health interventions.

**Table 3:** Study design characteristics of clinical trials that evaluated the efficacy of AI-based DHIs.

Author	Control Arm	Parallel Design	Randomization	Blinding	Multiple Countries	Multicenter	Powering Analysis	Dropout (<20%)	Long-Term Follow-Up (≥12 Weeks)
Metabolic Disorders									
Alfonsi [12]	+	+	+	NR	-	-	+	+	+
Avari [13]	+	-	+	-	+	+	+	+	+
Benhamou [14]	+	-	+	-	-	+	+	+	+
Biester [15]	+	-	+	-	+	+	+	-	-
Castle [16]	-	-	-	-	-	-	-	+	-
Jacobs [17]	+	-	+	-	-	-	+	+	-
Mosquera-Lopez [18]	+	-	+	-	-	-	-	-	-
Nimri [19]	+	+	+	+	+	+	+	+	+
Kannenberg [20]	-	-	-	-	-	-	-	-	+
Lee [21]	+	+	+	-	-	+	+	+	+
Nayak [22]	+	+	+	-	-	+	+	-	-
Ruiz-Leon [23]	+	+	+	+	-	+	+	+	+
Turnin [24]	+	+	+	-	-	+	+	+	+
Kelly & Holt [25]	+	+	+	NR	-	+	NR	NR	+
Kelly & Price [26]	+	+	+	NR	-	+	NR	NR	+
Caballero-Ruiz [27]	+	+	+	NR	-	-	NR	+	-
Choi [28]	+	+	+	NR	-	NR	+	+	+
Forman & Zhang [29]	-	-	-	-	-	NR	-	+	-
Forman & Crochiere [30]	+	+	+	-	-	NR	+	+	-
Goldstein [31]	+	+	+	NR	-	NR	+	+	-
Hassoon [32]	+	+	+	-	-	NR	+	+	-
Nakata [33]	+	+	+	-	-	+	+	+	+
Popp [34]	+	+	+	NR	-	-	+	-	+
Zarkogianni [35]	+	+	+	-	-	-	+	NR	+
Musculoskeletal Disorders									
Anan [36]	+	+	+	-	-	-	NR	+	+
Itoh [37]	+	+	+	-	-	+	+	+	+
Marcuzzi, 2024 [38]	+	+	+	-	-	-	+	+	+
Marcuzzi, 2023 [39]	-	-	-	-	-	-	NR	-	+
Nordstoga [40]	+	+	+	NR	+	+	NR	-	+
Park [41]	+	+	+	+	-	NR	+	NR	-
Piette [42]	+	+	+	-	-	+	+	+	+
Priebe [43]	+	+	+	NR	-	NR	+	-	+
Sandal, 2021 [44]	-	-	-	-	+	+	-	-	-
Sandal, 2020 [45]	+	+	+	-	+	+	+	+	+
Xiao [46]	+	+	+	-	-	-	+	+	-
Pelle, 2020 [47]	+	+	+	-	-	-	+	-	+
Pelle, 2021 [48]	-	-	-	-	-	-	-	-	+
Li, 2023 [49]	+	+	+	-	-	+	+	-	+
Davison [50]	-	-	-	-	-	-	-	-	-
Ortiz-Catalan [51]	-	-	-	-	+	+	-	+	+
He [52]	+	+	+	-	-	-	+	+	+
Mental Health Disorders									
Bricker [53]	+	+	+	+	-	-	-	+	+
Masaki [54]	+	+	+	+	-	NR	+	+	+
Olano-Espinosa [55]	+	+	+	-	-	+	+	-	+
Schnall [56]	+	+	+	-	-	+	-	+	+
Campellone [57]	+	+	+	+	-	+	-	+	-
Danieli, 2022 [58]	+	+	+	NR	-	NA	NR	+	+
Sandkuhler [11]	+	+	+	+	-	NR	NR	NR	NR
Browning [59]	+	+	+	-	+	+	+	-	+
Burns [60]	-	-	-	-	-	NR	NR	+	-
Danieli, 2021 [61]	+	+	+	NR	-	NA	NR	-	+
Fulmer [62]	+	+	+	-	-	NR	NR	+	-
Furukawa [63]	+	+	+	-	-	NR	NR	+	+
Liu [64]	+	+	+	-	-	+	+	-	+
Wang [65]	+	+	+	NR	-	+	+	+	-
Suharwardy [66]	+	+	+	-	-	-	+	-	-
Dimeff [67]	+	+	+	-	-	+	NR	+	-
Cardiovascular Disorders									
Gelman [68]	-	-	-	-	-	-	-	+	-
Yoon [69]	+	+	+	-	-	+	+	+	-
Persell [70]	+	+	+	-	-	+	+	+	+
Tsoumpa [71]	+	+	+	-	-	-	+	+	-
Wijnberge [72]	+	+	+	NR	-	-	+	+	-
Cerebrovascular Disorders									
Aguirre-Ollinger [73]	-	-	-	-	-	-	-	+	+
Chae [74]	+	+	+	NR	-	+	+	-	+
Kim [75]	+	+	+	-	-	NR	+	+	-
Labovitz [76]	+	+	+	NR	-	NR	NR	+	+
Pulmonary Disorders									
Seol [77]	+	+	+	+	-	+	+	NR	+
Silberman [78]	+	+	+	-	-	NR	+	-	+
Turino [79]	+	+	+	-	-	-	+	+	+
Neurodevelopmental Disorders									
Aydemir [80]	+	+	+	-	-	-	NR	NR	-
Daniels [81]	-	-	-	-	-	-	NR	+	-
Voss [82]	+	-	+	-	-	NR	-	-	+
Oncological Disorders									
Hu [83]	+	+	+	-	-	-	+	+	+
Li, 2025 [84]	+	+	+	+	-	-	+	+	+
Gastrointestinal Disorders									
Jactel [85]	-	-	-	-	-	-	+	+	-
Rafferty [86]	+	+	+	NR	-	-	+	-	-
Central Nervous System Disorders									
Schmitter-Edgecombe [87]	+	+	+	-	-	-	+	+	+
Pach [88]	+	+	+	NR	-	-	+	+	+
Other Disorders									
Meijer [89]	+	+	+	+	-	+	+	+	-
Zhu [90]	+	+	+	NR	-	NR	NR	NR	-
Ogawa [91]	+	+	+	NR	-	-	NR	+	+
Barakat-Johnson [92]	-	-	-	-	-	+	-	+	+
Holekamp [93]	-	-	-	-	-	+	NR	+	+
Li, 2024 [94]	+	+	+	+	-	-	+	+	+

Abbreviations: NA—Not applicable; NR—Not reported. Captions: The sign ‘+’ was used to present studies that fulfilled the respective criteria. The sign ‘-’ was used to present studies that did not fulfil the respective criteria.

**Table 4:** Additional study outcomes evaluated in the AI-based DHI clinical trials.

Author	Quality of Life	Safety	Economic Outcomes	Usability	Feasibility	Acceptance	Adherence	Satisfaction
Metabolic Disorders								
Alfonsi [12]	+	-	-	+	-	-	-	-
Avari [13]	+	+	-	-	-	-	-	-
Benhamou [14]	-	+	-	-	-	-	-	-
Biester [15]	-	-	-	-	-	-	-	-
Castle [16]	-	-	-	-	-	-	-	-
Jacobs [17]	-	+	-	-	-	-	-	-
Mosquera-Lopez [18]	-	-	-	-	-	-	-	-
Nimri [19]	-	+	-	-	-	-	-	+
Kannenberg [20]	+	-	-	-	-	-	-	+
Lee [21]	-	+	-	-	-	-	-	+
Nayak [22]	-	-	-	-	-	-	+	-
Ruiz-Leon [23]	+	-	-	-	-	-	-	-
Turnin [24]	-	-	-	-	-	-	+	+
Kelly & Holt [25]	+	-	+	-	-	-	-	-
Kelly & Price [26]	+	-	-	-	-	-	-	+
Caballero-Ruiz [27]	-	-	-	-	-	-	+	+
Choi [28]	-	-	-	-	-	-	-	-
Forman & Zhang [29]	-	-	-	-	-	+	-	-
Forman & Crochiere [30]	-	-	-	-	-	+	-	-
Goldstein [31]	-	-	-	-	-	-	+	-
Hassoon [32]	-	+	-	-	-	-	+	-
Nakata [33]	-	+	-	-	-	-	-	-
Popp [34]	-	-	-	-	-	-	+	-
Zarkogianni [35]	-	-	-	-	-	-	+	-
Musculoskeletal Disorders								
Anan [36]	-	-	-	-	-	-	+	-
Itoh [37]	+	-	-	+	-	-	+	-
Marcuzzi, 2024 [38]	+	+	-	-	-	-	-	-
Marcuzzi, 2023 [39]	-	-	-	+	-	-	+	-
Nordstoga [40]	-	-	-	-	-	-	-	-
Park [41]	+	+	+	-	-	-	-	+
Piette [42]	+	-	-	-	-	-	-	-
Priebe [43]	+	-	+	-	-	-	-	+
Sandal, 2021 [44]	+	-	-	-	-	-	-	-
Sandal, 2020 [45]	+	-	-	-	-	-	-	-
Xiao [46]	-	-	-	-	-	-	-	-
Pelle, 2020 [47]	+	-	+	-	-	-	-	-
Pelle, 2021 [48]	+	-	-	+	-	-	-	-
Li, 2023 [49]	+	+	-	-	-	-	+	-
Davison [50]	-	-	-	-	+	-	-	-
Ortiz-Catalan [51]	-	-	-	-	-	-	-	-
He [52]	+	-	-	-	-	-	-	-
Mental Health Disorders								
Bricker [53]	-	-	-	-	+	-	-	-
Masaki [54]	-	+	-	+	-	-	-	-
Olano-Espinosa [55]	+	-	-	-	-	-	-	-
Schnall [56]	-	-	-	+	-	-	-	-
Campellone [57]	-	+	-	+	-	-	-	+
Danieli, 2022 [58]	-	-	-	-	-	-	-	-
Sandkuhler [11]	-	-	-	-	-	-	-	-
Browning [59]	-	-	-	-	-	-	-	-
Burns [60]	-	-	-	-	-	-	-	-
Danieli, 2021 [61]	-	-	-	-	-	-	-	-
Fulmer [62]	-	-	-	-	-	-	-	+
Furukawa [63]	+	-	-	-	-	-	-	-
Liu [64]	-	-	-	-	-	-	+	+
Wang [65]	-	-	-	-	-	-	-	-
Suharwardy [66]	-	-	-	-	-	-	-	+
Dimeff [67]	-	+	-	-	+	+	-	+
Cardiovascular Disorders								
Gelman [68]	-	-	+	-	-	-	-	-
Yoon [69]	-	-	-	-	-	-	-	-
Persell [70]	-	-	-	-	-	-	+	-
Tsoumpa [71]	-	-	-	-	-	-	-	-
Wijnberge [72]	-	+	-	-	-	-	-	-
Cerebrovascular Disorders								
Aguirre-Ollinger [73]	+	+	+	-	-	+	+	-
Chae [74]	-	-	-	-	-	-	-	-
Kim [75]	+	-	-	+	-	-	-	-
Labovitz [76]	-	-	-	+	-	-	+	-
Pulmonary Disorders								
Seol [77]	-	+	+	-	-	-	-	-
Silberman [78]	-	-	-	-	-	-	+	-
Turino [79]	+	-	-	-	-	-	+	+
Neurodevelopmental Disorders								
Aydemir [80]	-	-	-	-	+	-	-	-
Daniels [81]	-	-	-	-	-	-	-	-
Voss [82]	-	-	-	-	-	-	-	-
Oncological Disorders								
Hu [83]	+	-	-	-	-	-	-	-
Li, 2025 [84]	+	+	-	-	-	-	+	-
Gastrointestinal Disorders								
Jactel [85]	+	-	-	-	-	-	+	-
Rafferty [86]	+	-	-	-	-	-	-	-
Central Nervous System Disorders								
Schmitter-Edgecombe [87]	+	-	-	-	-	-	-	+
Pach [88]	+	+	-	-	-	-	+	-
Other Disorders								
Meijer [89]	-	-	-	-	-	-	-	-
Zhu [90]	-	-	-	-	-	-	-	-
Ogawa [91]	-	-	-	-	-	-	-	-
Barakat-Johnson [92]	-	-	+	+	-	+	-	-
Holekamp [93]	-	-	+	-	-	-	-	-
Li, 2024 [94]	+	-	-	-	-	-	-	-

Captions: The sign ‘+’ was used to present studies that fulfilled the respective criteria. The sign ‘-’ was used to present studies that did not fulfil the respective criteria.

**Table 5:** Statistical procedures employed to assess study outcomes in AI-based DHI clinical trials.

Author	Regression Models	Normality Tests	Between-Group Comparison	Within-Group Comparison	Analysis of Variance	Analysis of Covariance
Metabolic Disorders						
Alfonsi [12]	+	-	+	-	-	-
Avari [13]	-	+	+	-	-	-
Benhamou [14]	+	-	-	-	-	-
Biester [15]	-	-	+	+	-	-
Castle [16]	+	-	+	-	-	-
Jacobs [17]	+	-	-	-	-	-
Mosquera-Lopez [18]	+	-	-	-	-	-
Nimri [19]	-	-	+	+	-	+
Kannenberg [20]	-	-	-	+	-	-
Lee [21]	-	-	+	-	+	-
Nayak [22]	-	-	+	-	-	-
Ruiz-Leon [23]	-	-	+	-	+	+
Turnin [24]	+	-	-	-	-	-
Kelly & Holt [25]	NR	NR	NR	NR	NR	NR
Kelly & Price [26]	NR	NR	NR	NR	NR	NR
Caballero-Ruiz [27]	-	-	+	-	-	-
Choi [28]	-	+	-	+	+	-
Forman & Zhang [29]	-	-	+	+	+	-
Forman & Crochiere [30]	+	-	+	-	+	+
Goldstein [31]	+	-	+	-	+	-
Hassoon [32]	+	-	-	-	-	-
Nakata [33]	-	-	+	-	-	+
Popp [34]	+	-	+	-	-	-
Zarkogianni [35]	-	+	+	+	-	-
Musculoskeletal Disorders						
Anan [36]	+	-	-	-	-	-
Itoh [37]	-	-	+	-	+	+
Marcuzzi, 2024 [38]	+	-	-	-	-	-
Marcuzzi, 2023 [39]	-	-	-	-	-	-
Nordstoga [40]	+	-	-	-	-	-
Park [41]	-	-	+	-	+	-
Piette [42]	+	-	-	-	-	-
Priebe [43]	+	-	-	-	+	-
Sandal, 2021 [44]	-	-	-	-	-	-
Sandal, 2020 [45]	+	-	-	-	-	-
Xiao [46]	+	+	+	-	-	+
Pelle, 2020 [47]	+	-	-	-	-	-
Pelle, 2021 [48]	-	-	+	-	-	-
Li, 2023 [49]	-	+	+	-	-	-
Davison [50]	+	-	-	-	-	-
Ortiz-Catalan [51]	-	-	+	-	-	-
He [52]	-	+	-	-	+	-
Mental Health Disorders						
Bricker [53]	+	-	+	-	+	-
Masaki [54]	+	-	-	-	-	+
Olano-Espinosa [55]	+	-	+	-	+	-
Schnall [56]	+	-	+	-	-	-
Campellone [57]	-	-	-	-	-	-
Danieli, 2022 [58]	-	-	-	-	+	-
Sandkuhler [11]	-	-	+	-	+	-
Browning [59]	+	-	-	-	-	-
Burns [60]	+	-	-	-	-	-
Danieli, 2021 [61]	-	-	+	-	-	-
Fulmer [62]	-	-	-	-	+	-
Furukawa [63]	+	-	-	-	-	-
Liu [64]	-	-	+	-	+	+
Wang [65]	-	-	+	-	+	-
Suharwardy [66]	-	-	+	-	-	-
Dimeff [67]	+	-	+	-	-	-
Cardiovascular Disorders						
Gelman [68]	-	-	+	-	-	-
Yoon [69]	+	-	-	-	-	-
Persell [70]	-	-	+	+	-	-
Tsoumpa [71]	-	+	+	-	-	-
Wijnberge [72]	-	+	+	-	-	-
Cerebrovascular Disorders						
Aguirre-Ollinger [73]	+	+	-	+	-	-
Chae [74]	-	-	+	-	-	-
Kim [75]	-	+	+	+	-	-
Labovitz [76]	NR	NR	NR	NR	NR	NR
Pulmonary Disorders						
Seol [77]	+	-	+	-	-	+
Silberman [78]	+	-	-	-	-	-
Turino [79]	+	-	+	-	-	-
Neurodevelopmental Disorders						
Aydemir [80]	-	-	-	-	+	-
Daniels [81]	-	-	+	-	+	-
Voss [82]	+	-	-	-	-	-
Oncological Disorders						
Hu [83]	-	-	+	+	-	-
Li, 2025 [84]	-	+	+	-	-	-
Gastrointestinal Disorders						
Jactel [85]	-	-	+	-	+	-
Rafferty [86]	-	-	+	-	-	-
Central Nervous System Disorders						
Schmitter-Edgecombe [87]	-	-	+	-	+	-
Pach [88]	+	-	+	-	-	+
Other Disorders						
Meijer [89]	+	-	+	-	-	-
Zhu [90]	-	-	+	-	-	-
Ogawa [91]	-	+	-	-	+	-
Barakat-Johnson [92]	+	-	-	-	-	-
Holekamp [93]	-	-	+	+	-	-
Li, 2024 [94]	+	-	-	-	+	-

Abbreviations: NR—Not reported. Captions: The sign ‘+’ was used to present studies that fulfilled the respective criteria. The sign ‘-’ was used to present studies that did not fulfil the respective criteria.

## Data Availability

No new data were created or analyzed in this study. Data sharing is not applicable to this article.

## Supplementary Materials

The following supporting information can be downloaded at: https://www.mdpi.com/article/10.3390/jmahp14030038/s1, Table S1: Search strings; Table S2: NICE quality appraisal checklist.
