# Supplementary material for: The Analytical Framework of Clinical Trials Evaluating Clinical Outcomes of Artificial Intelligence-Based Digital Health Interventions: A Systematic Literature Review
Source: J Mark Access Health Policy. 2026 Jul 1;14(3):38. doi: 10.3390/jmahp14030038 (PMC13398107; doi:10.3390/jmahp14030038)
Supplement: Supplementary file 1 [file jmahp-14-00038-s001.zip › jmahp-4364028-supplementary.pdf]

## Supplementary Materials

Table S1: Search strings

| Database | Query |
|---|---|
| **PubMed** | ("Digital Health"[MeSH Terms] OR "digital health intervention*"[All Fields] OR "digital intervention*"[All Fields] OR "digital health technolog*"[All Fields] OR "digital technolog*"[All Fields] OR "e-health"[All Fields] OR "m-health"[All Fields] OR "mobile health"[All Fields] OR "telehealth"[All Fields] OR "telemedicine"[All Fields] OR "digital medicine"[All Fields] OR "digital therapeutic*"[All Fields] OR "mobile medical app*"[All Fields] OR "virtual health"[All Fields] OR "remote health"[All Fields] OR "mobile health app*"[All Fields] OR "telemonitoring"[All Fields] OR "ehealth"[All Fields] OR "mhealth"[All Fields]) AND ("artificial intelligence"[MeSH Terms] OR ("artificial"[All Fields] AND "intelligence"[All Fields]) OR "artificial intelligence"[All Fields] OR "AI"[All Fields] OR "Artificial inteligence"[All Fields] OR "artificial intelligence"[MeSH Terms] OR "Machine Learning"[MeSH Terms] OR "Machine Learning"[All Fields] OR "deep learning"[All Fields] OR "AI"[All Fields]) AND (((("Clinical Trial"[Publication Type] NOT "protocol"[All Fields]) NOT ("Animals"[MeSH Terms] NOT ("Animals"[MeSH Terms] AND "Humans"[MeSH Terms]))) AND "english"[Language]) NOT ("pubmed books"[Filter] OR "editorial"[Publication Type] OR "guideline"[Publication Type] OR "interview"[Publication Type] OR "letter"[Publication Type] OR "practice guideline"[Publication Type] OR "review"[Publication Type] OR "systematic review"[Filter] OR "systematic review"[All Fields] OR "systematic literature review"[All Fields] OR "scoping review"[All Fields] OR "scoping literature review"[All Fields] OR "narrative review"[All Fields] OR "umbrella review"[All Fields] OR "realistic review"[All Fields] OR "narrative literature review"[All Fields] OR "umbrella literature review"[All Fields] OR "meta-analysis"[All Fields] OR "meta-analysis"[All Fields])) |
| **Embase** | ('digital health technology'/syn OR 'digital health technologies' OR 'digital technology' OR 'digital technologies' OR 'digital intervention'/syn OR 'digital health intervention'/syn OR 'digital health interventions' OR 'digital interventions' OR 'digital health'/syn OR 'ehealth' OR 'e-health' OR 'telecare'/syn OR 'mhealth'/syn OR 'm-health' OR 'mobile health' OR 'telehealth'/syn OR 'telemedicine' OR 'digital medicine' OR 'digital therapeutic' OR 'digital therapeutics' OR 'mobile health application'/syn OR 'mobile medical application' OR 'mobile medical app' OR 'mobile medical applications' OR 'mobile medical apps' OR 'mobile health app' OR 'mobile health application' OR 'virtual health' OR 'remote health' OR 'telemonitoring') AND ('artificial intelligence'/syn OR 'artificial inteligence' OR 'machine learning'/syn OR 'deep learning'/syn OR 'ai') AND [english]/lim AND ('adaptive clinical trial'/de OR 'adaptive clinical trial topic'/de OR 'clinical article'/de OR 'clinical study'/de OR 'clinical trial'/de OR 'clinical trial topic'/de OR 'controlled clinical trial'/de OR 'controlled clinical trial topic'/de OR 'diagnostic test accuracy study'/de OR 'equivalence trial'/de OR 'multicenter study'/de OR 'non inferiority trial'/de OR 'phase 1 clinical trial'/de OR 'phase 1 clinical trial topic'/de OR 'phase 2 clinical trial'/de OR 'phase 2 clinical trial topic'/de OR 'phase 3 clinical trial'/de OR 'phase 3 clinical trial topic'/de OR 'phase 4 clinical trial'/de OR 'phase 4 clinical trial topic'/de OR 'randomized controlled trial'/de OR 'randomized controlled trial topic'/de) NOT ('protocol' OR 'literature review' OR 'systematic review' OR 'slr' OR 'narrative review' OR 'scoping review' OR 'umbrella review' OR 'meta-analysis') AND ('article'/it OR 'article in press'/it) |

Table S2: NICE quality appraisal checklist

| Study Identification (First Author and Publication Year) | Aguirre-Ollinger 2024 | Alfonsi 2020 | Anan 2021 | Avari 2021 | Aydemir 2025 | Barakat-Johnson 2022 | Benhamou 2019 | Biester 2018 | Bricker 2024 | Browning 2021 |
|---|---|---|---|---|---|---|---|---|---|---|
| **Section 1: Population** | | | | | | | | | | |
| **1.1 Is the source population or source area well described?** | ++ | ++ | ++ | ++ | ++ | ++ | ++ | ++ | ++ | ++ |
| **1.2 Is the eligible population or area representative of the source population or area?** | ++ | ++ | ++ | ++ | + | + | ++ | ++ | ++ | ++ |
| **1.3 Do the selected participants or areas represent the eligible population or area?** | + | + | + | + | + | ++ | ++ | ++ | ++ | ++ |
| **Section 2: Method of allocation to intervention (or comparison)** | | | | | | | | | | |
| **2.1 Allocation to intervention (or comparison). How was selection bias minimized?** | NA | ++ | ++ | ++ | ++ | NA | ++ | ++ | ++ | ++ |

| | | | | | | | | | | |
|---|---|---|---|---|---|---|---|---|---|---|
| **2.2 Were interventions (and comparisons) well described and appropriate?** | ++ | + | ++ | ++ | + | ++ | ++ | ++ | ++ | ++ |
| **2.3 Was the allocation concealed?** | NA | ++ | ++ | ++ | ++ | NA | ++ | ++ | ++ | ++ |
| **2.4 Were participants or investigators blind to exposure and comparison?** | NA | NR | + | + | + | NA | + | + | ++ | + |
| **2.5 Was the exposure to the intervention and comparison adequate?** | ++ | ++ | + | ++ | ++ | ++ | ++ | ++ | ++ | ++ |
| **2.6 Was contamination acceptably low?** | NA | ++ | ++ | ++ | ++ | NA | ++ | ++ | ++ | ++ |
| **2.7 Were other interventions similar in both groups?** | NA | ++ | + | ++ | ++ | NA | ++ | ++ | ++ | ++ |
| **2.8 Were all participants accounted for at the study conclusion?** | ++ | ++ | + | ++ | ++ | ++ | ++ | + | + | + |
| **2.9 Did the setting reflect usual UK practice?** | ++ | ++ | ++ | ++ | ++ | ++ | ++ | ++ | ++ | ++ |
| **2.10 Did the intervention or control comparison reflect usual UK practice?** | ++ | ++ | ++ | ++ | ++ | | ++ | ++ | ++ | ++ |
| **Section 3: Outcomes** | | | | | | | | | | |
| **3.1 Were outcome measures reliable?** | ++ | ++ | + | ++ | + | ++ | ++ | ++ | ++ | ++ |
| **3.2 Were all outcome measurements complete?** | ++ | ++ | ++ | ++ | ++ | ++ | ++ | ++ | ++ | ++ |
| **3.3 Were all important outcomes assessed?** | ++ | ++ | ++ | ++ | ++ | ++ | ++ | ++ | ++ | ++ |
| **3.4 Were outcomes relevant?** | ++ | ++ | ++ | ++ | ++ | ++ | ++ | ++ | ++ | ++ |
| **3.5 Were there similar follow-up times in exposure and comparison groups?** | NA | ++ | ++ | ++ | ++ | NA | ++ | ++ | ++ | ++ |
| **3.6 Was follow-up time meaningful?** | ++ | ++ | ++ | ++ | + | ++ | ++ | ++ | ++ | ++ |
| **Section 4: Analyses** | | | | | | | | | | |
| **4.1 Were exposure and comparison groups similar at baseline? If not, were these adjusted?** | NA | ++ | + | ++ | ++ | NA | ++ | ++ | ++ | ++ |
| **4.2 Was the intention to treat (ITT) analysis conducted?** | ++ | ++ | ++ | ++ | ++ | ++ | ++ | ++ | ++ | ++ |
| **4.3 Was the study sufficiently powered to detect an intervention effect (if one exists)?** | NA | ++ | NR | ++ | NR | NA | ++ | ++ | NA | ++ |
| **4.4 Were the estimates of effect size given or calculable?** | ++ | ++ | ++ | ++ | ++ | ++ | ++ | ++ | ++ | ++ |
| **4.5 Were the analytical methods appropriate?** | ++ | ++ | ++ | ++ | ++ | + | ++ | ++ | ++ | ++ |
| **4.6 Was the precision of intervention effects given or calculable? Were they meaningful?** | ++ | ++ | ++ | ++ | ++ | ++ | ++ | ++ | ++ | ++ |
| **Section 5: Summary** | | | | | | | | | | |
| **5.1 Are the study results internally valid (i.e., unbiased)?** | ++ | ++ | + | ++ | + | + | ++ | ++ | ++ | ++ |
| **5.2 Are the findings generalizable to the source population (i.e., externally valid)?** | ++ | ++ | + | ++ | + | + | ++ | ++ | + | ++ |

Table S2: NICE quality appraisal checklist (continued)

| **Study Identification (First Author and Publication Year)** | Burns 2011 | Caballero-Ruiz 2017 | Campellone 2025 | Castle 2022 | Chae 2020 | Choi 2023 | Danieli 2021 | Danieli 2022 | Daniels 2018 | Davison 2024 |
|---|---|---|---|---|---|---|---|---|---|---|
| **Section 1: Population** | | | | | | | | | | |
| **1.1 Is the source population or source area well described?** | ++ | + | ++ | ++ | ++ | ++ | ++ | ++ | ++ | ++ |
| **1.2 Is the eligible population or area representative of the source population or area?** | + | + | ++ | + | ++ | ++ | + | + | + | + |
| **1.3 Do the selected participants or areas represent the eligible population or area?** | ++ | ++ | ++ | ++ | ++ | ++ | ++ | + | ++ | + |
| **Section 2: Method of allocation to intervention (or comparison)** | | | | | | | | | | |
| **2.1 Allocation to intervention (or comparison). How was selection bias minimized?** | NA | ++ | ++ | NA | ++ | ++ | ++ | ++ | NA | NA |
| **2.2 Were interventions (and comparisons) well described and appropriate?** | ++ | + | ++ | ++ | ++ | ++ | ++ | ++ | ++ | ++ |
| **2.3 Was the allocation concealed?** | NA | ++ | ++ | NA | ++ | ++ | ++ | ++ | NA | NA |
| **2.4 Were participants or investigators blind to exposure and comparison?** | NA | NR | ++ | NA | NR | NR | NR | NR | NA | NA |
| **2.5 Was the exposure to the intervention and comparison adequate?** | ++ | ++ | ++ | ++ | ++ | ++ | ++ | ++ | ++ | ++ |
| **2.6 Was contamination acceptably low?** | NA | ++ | ++ | NA | ++ | ++ | ++ | ++ | NA | NA |
| **2.7 Were other interventions similar in both groups?** | NA | ++ | ++ | NA | ++ | ++ | ++ | ++ | NA | NA |
| **2.8 Were all participants accounted for at the study conclusion?** | ++ | ++ | ++ | ++ | + | ++ | + | ++ | ++ | + |
| **2.9 Did the setting reflect usual UK practice?** | ++ | ++ | ++ | ++ | ++ | ++ | ++ | ++ | ++ | ++ |
| **2.10 Did the intervention or control comparison reflect usual UK practice?** | ++ | ++ | ++ | ++ | ++ | ++ | ++ | ++ | ++ | ++ |
| **Section 3: Outcomes** | | | | | | | | | | |
| **3.1 Were outcome measures reliable?** | ++ | ++ | ++ | ++ | ++ | ++ | ++ | ++ | ++ | ++ |
| **3.2 Were all outcome measurements complete?** | ++ | ++ | ++ | ++ | ++ | ++ | ++ | ++ | ++ | ++ |
| **3.3 Were all important outcomes assessed?** | ++ | + | ++ | ++ | ++ | ++ | ++ | ++ | ++ | ++ |
| **3.4 Were outcomes relevant?** | ++ | ++ | ++ | ++ | ++ | ++ | ++ | ++ | ++ | ++ |

| | | | | | | | | | | |
|---|---|---|---|---|---|---|---|---|---|---|
| 3.5 Were there similar follow-up times in exposure and comparison groups? | NA | ++ | ++ | NA | ++ | ++ | ++ | ++ | NA | NA |
| 3.6 Was follow-up time meaningful? | ++ | ++ | ++ | ++ | ++ | ++ | ++ | ++ | ++ | ++ |
| Section 4: Analyses | | | | | | | | | | |
| 4.1 Were exposure and comparison groups similar at baseline? If not, were these adjusted? | ++ | ++ | ++ | NA | + | ++ | ++ | ++ | ++ | ++ |
| 4.2 Was the intention to treat (ITT) analysis conducted? | ++ | ++ | ++ | ++ | ++ | ++ | ++ | ++ | ++ | ++ |
| 4.3 Was the study sufficiently powered to detect an intervention effect (if one exists)? | NA | NR | + | ++ | ++ | ++ | NA | NR | NA | NA |
| 4.4 Were the estimates of effect size given or calculable? | ++ | ++ | ++ | ++ | ++ | ++ | ++ | ++ | ++ | ++ |
| 4.5 Were the analytical methods appropriate? | ++ | + | ++ | ++ | ++ | ++ | ++ | ++ | ++ | ++ |
| 4.6 Was the precision of intervention effects given or calculable? Were they meaningful? | ++ | ++ | ++ | ++ | ++ | ++ | ++ | ++ | ++ | ++ |
| Section 5: Summary | | | | | | | | | | |
| 5.1 Are the study results internally valid (i.e., unbiased)? | + | + | ++ | + | + | + | + | + | + | + |
| 5.2 Are the findings generalizable to the source population (i.e., externally valid)? | + | + | + | ++ | ++ | ++ | ++ | ++ | + | + |

Table S2: NICE quality appraisal checklist (continued)

| Study Identification (First Author and Publication Year) | Dimeff 2022 | Forman 2019 | Forman 2019 | Fulmer 2018 | Furukawa 2025 | Gelman 2023 | Goldstein 2020 | Hassoon 2021 | He 2024 | Holekamp 2024 |
|---|---|---|---|---|---|---|---|---|---|---|
| Section 1: Population | | | | | | | | | | |
| 1.1 Is the source population or source area well described? | ++ | ++ | ++ | ++ | ++ | ++ | ++ | ++ | ++ | ++ |
| 1.2 Is the eligible population or area representative of the source population or area? | + | + | ++ | ++ | ++ | + | ++ | ++ | ++ | + |
| 1.3 Do the selected participants or areas represent the eligible population or area? | ++ | + | ++ | + | + | ++ | ++ | ++ | ++ | ++ |
| Section 2: Method of allocation to intervention (or comparison) | | | | | | | | | | |
| 2.1 Allocation to intervention (or comparison). How was selection bias minimized? | ++ | NA | ++ | ++ | ++ | NA | ++ | ++ | ++ | NA |
| 2.2 Were interventions (and comparisons) well described and appropriate? | ++ | ++ | ++ | + | ++ | ++ | ++ | ++ | ++ | ++ |
| 2.3 Was the allocation concealed? | ++ | NA | ++ | ++ | ++ | NA | ++ | ++ | ++ | NA |
| 2.4 Were participants or investigators blind to exposure and comparison? | + | NA | + | ++ | + | NA | NR | ++ | + | NA |
| 2.5 Was the exposure to the intervention and comparison adequate? | ++ | ++ | ++ | ++ | ++ | ++ | ++ | ++ | ++ | ++ |
| 2.6 Was contamination acceptably low? | ++ | NA | ++ | ++ | ++ | NA | ++ | ++ | ++ | NA |
| 2.7 Were other interventions similar in both groups? | ++ | NA | ++ | ++ | ++ | NA | ++ | ++ | ++ | NA |
| 2.8 Were all participants accounted for at the study conclusion? | ++ | ++ | ++ | ++ | ++ | ++ | ++ | ++ | ++ | ++ |
| 2.9 Did the setting reflect usual UK practice? | ++ | ++ | ++ | ++ | ++ | ++ | ++ | ++ | ++ | ++ |
| 2.10 Did the intervention or control comparison reflect usual UK practice? | ++ | ++ | ++ | ++ | ++ | ++ | ++ | ++ | ++ | ++ |
| Section 3: Outcomes | | | | | | | | | | |
| 3.1 Were outcome measures reliable? | ++ | ++ | ++ | ++ | ++ | ++ | ++ | ++ | ++ | ++ |
| 3.2 Were all outcome measurements complete? | ++ | ++ | ++ | ++ | ++ | ++ | ++ | ++ | ++ | ++ |
| 3.3 Were all important outcomes assessed? | ++ | ++ | ++ | ++ | ++ | ++ | ++ | ++ | ++ | ++ |
| 3.4 Were outcomes relevant? | ++ | ++ | ++ | ++ | ++ | ++ | ++ | ++ | ++ | ++ |
| 3.5 Were there similar follow-up times in exposure and comparison groups? | ++ | NA | ++ | ++ | ++ | ++ | ++ | ++ | ++ | NA |
| 3.6 Was follow-up time meaningful? | ++ | ++ | ++ | ++ | ++ | ++ | ++ | ++ | ++ | ++ |
| Section 4: Analyses | | | | | | | | | | |
| 4.1 Were exposure and comparison groups similar at baseline? If not, were these adjusted? | ++ | ++ | ++ | ++ | ++ | ++ | ++ | ++ | ++ | ++ |
| 4.2 Was the intention to treat (ITT) analysis conducted? | ++ | ++ | ++ | ++ | ++ | ++ | ++ | ++ | ++ | ++ |
| 4.3 Was the study sufficiently powered to detect an intervention effect (if one exists)? | NA | NR | ++ | NR | NR | NR | + | ++ | ++ | NR |
| 4.4 Were the estimates of effect size given or calculable? | ++ | ++ | ++ | ++ | ++ | ++ | ++ | ++ | ++ | ++ |
| 4.5 Were the analytical methods appropriate? | ++ | ++ | ++ | ++ | ++ | ++ | ++ | ++ | ++ | ++ |
| 4.6 Was the precision of intervention effects given or calculable? Were they meaningful? | ++ | ++ | ++ | ++ | ++ | ++ | ++ | ++ | ++ | ++ |
| Section 5: Summary | | | | | | | | | | |
| 5.1 Are the study results internally valid (i.e., unbiased)? | + | + | ++ | ++ | ++ | + | ++ | ++ | ++ | + |
| 5.2 Are the findings generalizable to the source population (i.e., externally valid)? | ++ | ++ | ++ | ++ | ++ | ++ | ++ | ++ | ++ | ++ |

Table S2: NICE quality appraisal checklist (continued)

| Study Identification (First Author and Publication Year) | Hu 2025 | Itoh 2022 | Jacobs 2023 | Jactel 2022 | Kannenberg 2024 | Kelly 2025 (Cost-Effective…) | Kelly 2025 (Delivering…) | Kim 2023 | Labovitz, 2017 | Lee, 2023 |
|---|---|---|---|---|---|---|---|---|---|---|
| Section 1: Population | | | | | | | | | | |

| | | | | | | | | | | |
|---|---|---|---|---|---|---|---|---|---|---|
| **1.1 Is the source population or source area well described?** | ++ | ++ | ++ | ++ | ++ | ++ | ++ | ++ | ++ | ++ |
| **1.2 Is the eligible population or area representative of the source population or area?** | ++ | ++ | ++ | + | ++ | ++ | ++ | ++ | ++ | ++ |
| **1.3 Do the selected participants or areas represent the eligible population or area?** | ++ | ++ | ++ | ++ | + | + | + | ++ | ++ | ++ |
| **Section 2: Method of allocation to intervention (or comparison)** | | | | | | | | | | |
| **2.1 Allocation to intervention (or comparison). How was selection bias minimized?** | ++ | ++ | ++ | NA | NA | ++ | ++ | ++ | ++ | ++ |
| **2.2 Were interventions (and comparisons) well described and appropriate?** | ++ | ++ | ++ | ++ | ++ | + | + | ++ | ++ | ++ |
| **2.3 Was the allocation concealed?** | ++ | ++ | ++ | NA | NA | ++ | ++ | ++ | ++ | ++ |
| **2.4 Were participants or investigators blind to exposure and comparison?** | ++ | ++ | + | NA | NA | NR | NR | ++ | NR | - |
| **2.5 Was the exposure to the intervention and comparison adequate?** | ++ | ++ | ++ | ++ | ++ | ++ | ++ | ++ | ++ | ++ |
| **2.6 Was contamination acceptably low?** | ++ | ++ | ++ | NA | NA | ++ | ++ | ++ | ++ | ++ |
| **2.7 Were other interventions similar in both groups?** | ++ | ++ | ++ | NA | NA | ++ | ++ | ++ | ++ | ++ |
| **2.8 Were all participants accounted for at the study conclusion?** | ++ | ++ | ++ | ++ | + | NR | NR | ++ | ++ | ++ |
| **2.9 Did the setting reflect usual UK practice?** | ++ | ++ | ++ | ++ | ++ | ++ | ++ | ++ | ++ | + |
| **2.10 Did the intervention or control comparison reflect usual UK practice?** | ++ | ++ | ++ | ++ | ++ | ++ | ++ | ++ | ++ | + |
| **Section 3: Outcomes** | | | | | | | | | | |
| **3.1 Were outcome measures reliable?** | ++ | ++ | ++ | ++ | ++ | ++ | ++ | ++ | ++ | ++ |
| **3.2 Were all outcome measurements complete?** | ++ | ++ | ++ | ++ | ++ | ++ | ++ | ++ | ++ | ++ |
| **3.3 Were all important outcomes assessed?** | ++ | ++ | ++ | ++ | ++ | ++ | ++ | ++ | + | ++ |
| **3.4 Were outcomes relevant?** | ++ | ++ | ++ | ++ | ++ | ++ | ++ | ++ | ++ | ++ |
| **3.5 Were there similar follow-up times in exposure and comparison groups?** | ++ | ++ | ++ | NA | NA | ++ | ++ | ++ | ++ | ++ |
| **3.6 Was follow-up time meaningful?** | ++ | ++ | ++ | ++ | ++ | ++ | ++ | ++ | ++ | ++ |
| **Section 4: Analyses** | | | | | | | | | | |
| **4.1 Were exposure and comparison groups similar at baseline? If not, were these adjusted?** | ++ | ++ | ++ | NA | NA | ++ | ++ | ++ | ++ | ++ |
| **4.2 Was the intention to treat (ITT) analysis conducted?** | ++ | ++ | ++ | NR | ++ | ++ | ++ | ++ | NR | ++ |
| **4.3 Was the study sufficiently powered to detect an intervention effect (if one exists)?** | ++ | ++ | + | ++ | NR | NR | NA | ++ | NR | ++ |
| **4.4 Were the estimates of effect size given or calculable?** | ++ | ++ | ++ | ++ | ++ | ++ | ++ | ++ | NR | ++ |
| **4.5 Were the analytical methods appropriate?** | ++ | ++ | ++ | ++ | ++ | ++ | ++ | ++ | NR | ++ |
| **4.6 Was the precision of intervention effects given or calculable? Were they meaningful?** | ++ | ++ | ++ | ++ | ++ | ++ | ++ | ++ | NR | ++ |
| **Section 5: Summary** | | | | | | | | | | |
| **5.1 Are the study results internally valid (i.e., unbiased)?** | ++ | ++ | ++ | + | + | + | + | ++ | + | + |
| **5.2 Are the findings generalizable to the source population (i.e., externally valid)?** | ++ | ++ | ++ | ++ | ++ | ++ | ++ | ++ | + | + |

Table S2: NICE quality appraisal checklist (continued)

| **Study Identification (First Author and Publication Year)** | Li, 2023 | Li, 2025 | Li, 2024 | Liu, 2022 | Marcuzzi, 2024 | Marcuzzi, 2023 | Masaki, 2020 | Meijer, 2020 | Mosquera-Lopez, 2023 | Nakata, 2022 |
|---|---|---|---|---|---|---|---|---|---|---|
| **Section 1: Population** | | | | | | | | | | |
| **1.1 Is the source population or source area well described?** | ++ | ++ | ++ | ++ | ++ | ++ | ++ | ++ | ++ | + |
| **1.2 Is the eligible population or area representative of the source population or area?** | ++ | ++ | ++ | + | ++ | ++ | + | + | ++ | + |
| **1.3 Do the selected participants or areas represent the eligible population or area?** | ++ | + | ++ | ++ | ++ | ++ | ++ | ++ | + | + |
| **Section 2: Method of allocation to intervention (or comparison)** | | | | | | | | | | |
| **2.1 Allocation to intervention (or comparison). How was selection bias minimized?** | ++ | ++ | ++ | ++ | NA | ++ | ++ | ++ | ++ | ++ |
| **2.2 Were interventions (and comparisons) well described and appropriate?** | ++ | + | ++ | ++ | ++ | ++ | ++ | ++ | ++ | ++ |
| **2.3 Was the allocation concealed?** | ++ | ++ | ++ | ++ | NA | ++ | ++ | ++ | ++ | ++ |
| **2.4 Were participants or investigators blind to exposure and comparison?** | - | + | ++ | - | - | - | + | ++ | - | - |
| **2.5 Was the exposure to the intervention and comparison adequate?** | ++ | ++ | ++ | ++ | ++ | ++ | ++ | ++ | ++ | ++ |
| **2.6 Was contamination acceptably low?** | ++ | ++ | ++ | ++ | NA | ++ | ++ | ++ | - | ++ |
| **2.7 Were other interventions similar in both groups?** | ++ | ++ | ++ | ++ | NA | ++ | ++ | ++ | ++ | ++ |
| **2.8 Were all participants accounted for at the study conclusion?** | + | ++ | ++ | + | ++ | - | ++ | ++ | + | ++ |

| | | | | | | | | | | |
|---|---|---|---|---|---|---|---|---|---|---|
| 2.9 Did the setting reflect usual UK practice? | + | + | + | + | ++ | ++ | ++ | + | ++ | ++ |
| 2.10 Did the intervention or control comparison reflect usual UK practice? | + | + | + | + | ++ | ++ | ++ | + | ++ | ++ |
| Section 3: Outcomes | | | | | | | | | | |
| 3.1 Were outcome measures reliable? | ++ | ++ | ++ | ++ | + | ++ | ++ | ++ | ++ | ++ |
| 3.2 Were all outcome measurements complete? | ++ | ++ | ++ | ++ | ++ | ++ | ++ | ++ | ++ | ++ |
| 3.3 Were all important outcomes assessed? | ++ | ++ | ++ | ++ | + | ++ | ++ | ++ | ++ | ++ |
| 3.4 Were outcomes relevant? | ++ | ++ | ++ | ++ | + | ++ | ++ | ++ | ++ | ++ |
| 3.5 Were there similar follow-up times in exposure and comparison groups? | ++ | ++ | ++ | ++ | ++ | ++ | ++ | + | ++ | ++ |
| 3.6 Was follow-up time meaningful? | ++ | ++ | ++ | ++ | ++ | ++ | ++ | ++ | + | ++ |
| Section 4: Analyses | | | | | | | | | | |
| 4.1 Were exposure and comparison groups similar at baseline? If not, were these adjusted? | ++ | ++ | ++ | ++ | NA | ++ | ++ | ++ | ++ | ++ |
| 4.2 Was the intention to treat (ITT) analysis conducted? | ++ | ++ | ++ | ++ | NR | ++ | ++ | NR | ++ | ++ |
| 4.3 Was the study sufficiently powered to detect an intervention effect (if one exists)? | ++ | ++ | ++ | ++ | NR | ++ | ++ | ++ | - | ++ |
| 4.4 Were the estimates of effect size given or calculable? | ++ | ++ | ++ | ++ | NA | ++ | ++ | ++ | + | ++ |
| 4.5 Were the analytical methods appropriate? | ++ | ++ | ++ | ++ | + | ++ | ++ | ++ | + | ++ |
| 4.6 Was the precision of intervention effects given or calculable? Were they meaningful? | ++ | ++ | ++ | ++ | ++ | ++ | ++ | ++ | + | ++ |
| Section 5: Summary | | | | | | | | | | |
| 5.1 Are the study results internally valid (i.e., unbiased)? | + | ++ | ++ | + | + | + | ++ | ++ | + | + |
| 5.2 Are the findings generalizable to the source population (i.e., externally valid)? | + | + | + | + | ++ | ++ | ++ | ++ | + | + |

Table S2: NICE quality appraisal checklist (continued)

| Study Identification (First Author and Publication Year) | Nayak, 2023 | Nimri, 2020 | Nordstoga, 2023 | Ogawa, 2022 | Olano-Espinosa, 2022 | Ortiz-Catalan, 2016 | Pach, 2025 | Park, 2023 | Pelle, 2020 | Pelle, 2021 |
|---|---|---|---|---|---|---|---|---|---|---|
| Section 1: Population | | | | | | | | | | |
| 1.1 Is the source population or source area well described? | ++ | + | ++ | ++ | ++ | + | ++ | ++ | ++ | ++ |
| 1.2 Is the eligible population or area representative of the source population or area? | ++ | ++ | + | + | + | + | ++ | + | + | + |
| 1.3 Do the selected participants or areas represent the eligible population or area? | + | ++ | ++ | + | ++ | + | ++ | ++ | ++ | ++ |
| Section 2: Method of allocation to intervention (or comparison) | | | | | | | | | | |
| 2.1 Allocation to intervention (or comparison). How was selection bias minimized? | ++ | ++ | ++ | ++ | ++ | NA | ++ | ++ | ++ | NA |
| 2.2 Were interventions (and comparisons) well described and appropriate? | ++ | ++ | ++ | ++ | ++ | ++ | ++ | ++ | ++ | ++ |
| 2.3 Was the allocation concealed? | ++ | ++ | ++ | ++ | ++ | NA | ++ | ++ | ++ | NA |
| 2.4 Were participants or investigators blind to exposure and comparison? | - | + | NR | NR | - | - | - | + | - | - |
| 2.5 Was the exposure to the intervention and comparison adequate? | ++ | ++ | ++ | ++ | ++ | ++ | ++ | ++ | ++ | ++ |
| 2.6 Was contamination acceptably low? | ++ | ++ | ++ | ++ | ++ | NA | ++ | ++ | ++ | NA |
| 2.7 Were other interventions similar in both groups? | ++ | ++ | ++ | ++ | ++ | NA | ++ | ++ | ++ | NA |
| 2.8 Were all participants accounted for at the study conclusion? | - | ++ | - | ++ | - | ++ | ++ | ++ | - | - |
| 2.9 Did the setting reflect usual UK practice? | ++ | ++ | ++ | ++ | + | + | ++ | + | + | + |
| 2.10 Did the intervention or control comparison reflect usual UK practice? | ++ | ++ | ++ | ++ | + | + | ++ | + | + | + |
| Section 3: Outcomes | | | | | | | | | | |
| 3.1 Were outcome measures reliable? | ++ | ++ | ++ | ++ | ++ | ++ | ++ | ++ | ++ | ++ |
| 3.2 Were all outcome measurements complete? | ++ | ++ | ++ | ++ | ++ | ++ | ++ | ++ | ++ | ++ |
| 3.3 Were all important outcomes assessed? | ++ | ++ | + | + | ++ | ++ | ++ | ++ | ++ | ++ |
| 3.4 Were outcomes relevant? | ++ | ++ | ++ | ++ | ++ | ++ | ++ | ++ | ++ | ++ |
| 3.5 Were there similar follow-up times in exposure and comparison groups? | ++ | ++ | ++ | ++ | ++ | NA | ++ | ++ | ++ | NA |
| 3.6 Was follow-up time meaningful? | ++ | ++ | ++ | ++ | ++ | ++ | ++ | ++ | ++ | ++ |
| Section 4: Analyses | | | | | | | | | | |
| 4.1 Were exposure and comparison groups similar at baseline? If not, were these adjusted? | ++ | ++ | ++ | ++ | ++ | NA | ++ | ++ | ++ | NA |
| 4.2 Was the intention to treat (ITT) analysis conducted? | ++ | ++ | ++ | ++ | ++ | NR | ++ | ++ | ++ | NR |
| 4.3 Was the study sufficiently powered to detect an intervention effect (if one exists)? | ++ | ++ | NR | NR | ++ | NA | ++ | ++ | ++ | NA |
| 4.4 Were the estimates of effect size given or calculable? | ++ | ++ | ++ | ++ | ++ | NA | ++ | ++ | ++ | NA |
| 4.5 Were the analytical methods appropriate? | ++ | ++ | ++ | ++ | ++ | + | ++ | ++ | ++ | + |

| | | | | | | | | | | |
|---|---|---|---|---|---|---|---|---|---|---|
| **4.6 Was the precision of intervention effects given or calculable? Were they meaningful?** | ++ | ++ | ++ | ++ | ++ | + | ++ | ++ | ++ | + |
| **Section 5: Summary** | | | | | | | | | | |
| **5.1 Are the study results internally valid (i.e., unbiased)?** | + | ++ | + | + | + | + | + | ++ | + | + |
| **5.2 Are the findings generalizable to the source population (i.e., externally valid)?** | + | ++ | + | + | + | + | ++ | + | + | + |

Table S2: NICE quality appraisal checklist (continued)

| **Study Identification (First Author and Publication Year)** | Persell, 2020 | Piette, 2022 | Popp, 2022 | Priebe, 2024 | Rafferty, 2021 | Ruiz-Leon, 2025 | Sandal, 2021 | Sandal, 2020 | Sandkuhler, 2025 | Schmitter-Edgecombe, 2022 |
|---|---|---|---|---|---|---|---|---|---|---|
| **Section 1: Population** | | | | | | | | | | |
| **1.1 Is the source population or source area well described?** | ++ | ++ | ++ | ++ | ++ | ++ | ++ | ++ | ++ | + |
| **1.2 Is the eligible population or area representative of the source population or area?** | ++ | ++ | ++ | ++ | ++ | + | + | + | ++ | ++ |
| **1.3 Do the selected participants or areas represent the eligible population or area?** | ++ | ++ | ++ | ++ | + | ++ | ++ | ++ | ++ | + |
| **Section 2: Method of allocation to intervention (or comparison)** | | | | | | | | | | |
| **2.1 Allocation to intervention (or comparison). How was selection bias minimized?** | ++ | ++ | ++ | ++ | ++ | ++ | ++ | NA | ++ | ++ |
| **2.2 Were interventions (and comparisons) well described and appropriate?** | ++ | ++ | ++ | ++ | ++ | ++ | ++ | ++ | ++ | ++ |
| **2.3 Was the allocation concealed?** | ++ | ++ | ++ | ++ | ++ | ++ | ++ | NA | ++ | ++ |
| **2.4 Were participants or investigators blind to exposure and comparison?** | - | - | NR | NR | NR | + | - | - | ++ | NR |
| **2.5 Was the exposure to the intervention and comparison adequate?** | ++ | ++ | ++ | ++ | ++ | ++ | ++ | ++ | ++ | ++ |
| **2.6 Was contamination acceptably low?** | ++ | ++ | ++ | ++ | ++ | ++ | ++ | NA | ++ | ++ |
| **2.7 Were other interventions similar in both groups?** | ++ | ++ | ++ | ++ | ++ | ++ | ++ | NA | ++ | ++ |
| **2.8 Were all participants accounted for at the study conclusion?** | ++ | ++ | ++ | - | - | ++ | - | ++ | NR | ++ |
| **2.9 Did the setting reflect usual UK practice?** | ++ | ++ | ++ | ++ | ++ | + | + | + | ++ | ++ |
| **2.10 Did the intervention or control comparison reflect usual UK practice?** | ++ | ++ | ++ | ++ | ++ | + | + | + | ++ | ++ |
| **Section 3: Outcomes** | | | | | | | | | | |
| **3.1 Were outcome measures reliable?** | ++ | ++ | ++ | ++ | ++ | ++ | ++ | ++ | ++ | ++ |
| **3.2 Were all outcome measurements complete?** | ++ | ++ | ++ | ++ | ++ | ++ | ++ | ++ | ++ | ++ |
| **3.3 Were all important outcomes assessed?** | ++ | ++ | ++ | ++ | ++ | ++ | ++ | ++ | ++ | ++ |
| **3.4 Were outcomes relevant?** | ++ | ++ | ++ | ++ | ++ | ++ | ++ | ++ | ++ | ++ |
| **3.5 Were there similar follow-up times in exposure and comparison groups?** | ++ | ++ | ++ | ++ | ++ | ++ | ++ | NA | ++ | ++ |
| **3.6 Was follow-up time meaningful?** | ++ | ++ | ++ | ++ | ++ | ++ | ++ | ++ | ++ | ++ |
| **Section 4: Analyses** | | | | | | | | | | |
| **4.1 Were exposure and comparison groups similar at baseline? If not, were these adjusted?** | ++ | ++ | ++ | ++ | ++ | ++ | ++ | NA | ++ | ++ |
| **4.2 Was the intention to treat (ITT) analysis conducted?** | ++ | ++ | ++ | ++ | ++ | ++ | ++ | NR | ++ | ++ |
| **4.3 Was the study sufficiently powered to detect an intervention effect (if one exists)?** | ++ | ++ | ++ | ++ | ++ | ++ | ++ | NA | NR | ++ |
| **4.4 Were the estimates of effect size given or calculable?** | ++ | ++ | ++ | ++ | ++ | ++ | ++ | NA | ++ | ++ |
| **4.5 Were the analytical methods appropriate?** | ++ | ++ | ++ | ++ | ++ | ++ | ++ | ++ | ++ | ++ |
| **4.6 Was the precision of intervention effects given or calculable? Were they meaningful?** | ++ | ++ | ++ | ++ | ++ | ++ | ++ | ++ | ++ | ++ |
| **Section 5: Summary** | | | | | | | | | | |
| **5.1 Are the study results internally valid (i.e., unbiased)?** | + | + | + | + | + | ++ | + | + | ++ | + |
| **5.2 Are the findings generalizable to the source population (i.e., externally valid)?** | ++ | ++ | ++ | + | + | ++ | + | + | ++ | ++ |

Table S2: NICE quality appraisal checklist (continued)

| **Study Identification (First Author and Publication Year)** | Schnall, 2025 | Seol, 2021 | Silberman, 2025 | Suharwardy, 2023 | Tsoumpa, 2021 | Turino, 2021 | Turnin, 2021 | Voss 2019 | Wang 2025 | Wijnberge 2020 |
|---|---|---|---|---|---|---|---|---|---|---|
| **Section 1: Population** | | | | | | | | | | |
| **1.1 Is the source population or source area well described?** | + | ++ | ++ | + | ++ | ++ | ++ | ++ | ++ | ++ |
| **1.2 Is the eligible population or area representative of the source population or area?** | ++ | ++ | ++ | ++ | + | + | + | ++ | ++ | ++ |
| **1.3 Do the selected participants or areas represent the eligible population or area?** | + | ++ | ++ | ++ | ++ | ++ | ++ | + | ++ | ++ |
| **Section 2: Method of allocation to intervention (or comparison)** | | | | | | | | | | |

| | | | | | | | | | | |
|---|---|---|---|---|---|---|---|---|---|---|
| 2.1 Allocation to intervention (or comparison). How was selection bias minimized? | ++ | ++ | ++ | ++ | ++ | ++ | ++ | ++ | ++ | ++ |
| 2.2 Were interventions (and comparisons) well described and appropriate? | ++ | ++ | ++ | ++ | ++ | ++ | ++ | ++ | ++ | ++ |
| 2.3 Was the allocation concealed? | ++ | ++ | ++ | ++ | ++ | ++ | ++ | ++ | ++ | ++ |
| 2.4 Were participants or investigators blind to exposure and comparison? | - | + | - | - | NR | - | - | + | NR | + |
| 2.5 Was the exposure to the intervention and comparison adequate? | ++ | ++ | ++ | ++ | ++ | ++ | ++ | ++ | ++ | ++ |
| 2.6 Was contamination acceptably low? | ++ | ++ | ++ | ++ | ++ | ++ | ++ | ++ | ++ | ++ |
| 2.7 Were other interventions similar in both groups? | ++ | ++ | ++ | ++ | ++ | ++ | ++ | ++ | ++ | ++ |
| 2.8 Were all participants accounted for at the study conclusion? | ++ | NR | - | ++ | ++ | ++ | ++ | + | ++ | ++ |
| 2.9 Did the setting reflect usual UK practice? | ++ | ++ | ++ | ++ | + | + | + | ++ | ++ | ++ |
| 2.10 Did the intervention or control comparison reflect usual UK practice? | ++ | ++ | ++ | ++ | + | + | + | ++ | ++ | ++ |
| **Section 3: Outcomes** | | | | | | | | | | |
| 3.1 Were outcome measures reliable? | ++ | ++ | ++ | ++ | ++ | ++ | ++ | ++ | ++ | ++ |
| 3.2 Were all outcome measurements complete? | ++ | ++ | ++ | ++ | ++ | ++ | ++ | ++ | ++ | ++ |
| 3.3 Were all important outcomes assessed? | ++ | ++ | ++ | ++ | ++ | ++ | ++ | ++ | ++ | ++ |
| 3.4 Were outcomes relevant? | ++ | ++ | ++ | ++ | ++ | ++ | ++ | ++ | ++ | ++ |
| 3.5 Were there similar follow-up times in exposure and comparison groups? | ++ | ++ | ++ | ++ | ++ | ++ | ++ | ++ | ++ | ++ |
| 3.6 Was follow-up time meaningful? | ++ | ++ | ++ | ++ | ++ | ++ | ++ | ++ | ++ | ++ |
| **Section 4: Analyses** | | | | | | | | | | |
| 4.1 Were exposure and comparison groups similar at baseline? If not, were these adjusted? | ++ | ++ | ++ | ++ | ++ | ++ | ++ | ++ | ++ | ++ |
| 4.2 Was the intention to treat (ITT) analysis conducted? | ++ | NR | ++ | ++ | ++ | ++ | ++ | ++ | ++ | NR |
| 4.3 Was the study sufficiently powered to detect an intervention effect (if one exists)? | ++ | ++ | ++ | ++ | ++ | ++ | ++ | ++ | ++ | ++ |
| 4.4 Were the estimates of effect size given or calculable? | ++ | ++ | ++ | ++ | ++ | ++ | ++ | ++ | ++ | ++ |
| 4.5 Were the analytical methods appropriate? | ++ | ++ | ++ | ++ | ++ | ++ | ++ | ++ | ++ | ++ |
| 4.6 Was the precision of intervention effects given or calculable? Were they meaningful? | ++ | ++ | ++ | ++ | ++ | ++ | ++ | ++ | ++ | ++ |
| **Section 5: Summary** | | | | | | | | | | |
| 5.1 Are the study results internally valid (i.e., unbiased)? | + | ++ | + | + | + | + | + | ++ | ++ | ++ |
| 5.2 Are the findings generalizable to the source population (i.e., externally valid)? | + | ++ | ++ | ++ | + | + | + | ++ | ++ | ++ |

Table S2: NICE quality appraisal checklist (continued)

| Study Identification (First Author and Publication Year) | Xiao 2025 | Yoon, 2024 | Zarkogianni, 2023 | Zhu, 2022 |
|---|---|---|---|---|
| **Section 1: Population** | | | | |
| 1.1 Is the source population or source area well described? | ++ | ++ | + | ++ |
| 1.2 Is the eligible population or area representative of the source population or area? | ++ | + | + | ++ |
| 1.3 Do the selected participants or areas represent the eligible population or area? | ++ | ++ | + | ++ |
| **Section 2: Method of allocation to intervention (or comparison)** | | | | |
| 2.1 Allocation to intervention (or comparison). How was selection bias minimized? | ++ | ++ | ++ | NR |
| 2.2 Were interventions (and comparisons) well described and appropriate? | ++ | ++ | ++ | + |
| 2.3 Was the allocation concealed? | ++ | ++ | ++ | NR |
| 2.4 Were participants or investigators blind to exposure and comparison? | + | - | - | NR |
| 2.5 Was the exposure to the intervention and comparison adequate? | ++ | ++ | ++ | + |
| 2.6 Was contamination acceptably low? | ++ | ++ | ++ | + |
| 2.7 Were other interventions similar in both groups? | ++ | ++ | ++ | + |
| 2.8 Were all participants accounted for at the study conclusion? | ++ | ++ | NR | NR |
| 2.9 Did the setting reflect usual UK practice? | ++ | + | + | + |
| 2.10 Did the intervention or control comparison reflect usual UK practice? | ++ | + | + | + |
| **Section 3: Outcomes** | | | | |
| 3.1 Were outcome measures reliable? | ++ | ++ | ++ | + |
| 3.2 Were all outcome measurements complete? | ++ | ++ | ++ | + |
| 3.3 Were all important outcomes assessed? | ++ | ++ | ++ | + |
| 3.4 Were outcomes relevant? | ++ | ++ | ++ | + |
| 3.5 Were there similar follow-up times in exposure and comparison groups? | ++ | ++ | ++ | + |
| 3.6 Was follow-up time meaningful? | ++ | ++ | ++ | + |
| **Section 4: Analyses** | | | | |

| | | | | |
|---|---|---|---|---|
| **4.1 Were exposure and comparison groups similar at baseline? If not, were these adjusted?** | ++ | ++ | ++ | ++ |
| **4.2 Was the intention to treat (ITT) analysis conducted?** | NR | ++ | ++ | NR |
| **4.3 Was the study sufficiently powered to detect an intervention effect (if one exists)?** | ++ | ++ | ++ | NR |
| **4.4 Were the estimates of effect size given or calculable?** | ++ | ++ | ++ | + |
| **4.5 Were the analytical methods appropriate?** | ++ | ++ | ++ | + |
| **4.6 Was the precision of intervention effects given or calculable? Were they meaningful?** | ++ | ++ | ++ | + |
| **Section 5: Summary** | | | | |
| **5.1 Are the study results internally valid (i.e., unbiased)?** | ++ | + | + | + |
| **5.2 Are the findings generalizable to the source population (i.e., externally valid)?** | ++ | ++ | + | + |
